# Description, microhabitat selection and infection patterns of sealworm larvae (*Pseudoterranova decipiens* species complex, nematoda: ascaridoidea) in fishes from Patagonia, Argentina

**DOI:** 10.1186/1756-3305-6-252

**Published:** 2013-08-29

**Authors:** Jesús S Hernández-Orts, Francisco J Aznar, Isabel Blasco-Costa, Néstor A García, María Víllora-Montero, Enrique A Crespo, Juan A Raga, Francisco E Montero

**Affiliations:** 1 Cavanilles Institute of Biodiversity and Evolutionary Biology, Science Park, University of Valencia, C/Catedrático José Beltrán 2, 46980, Paterna Valencia, Spain; 2 Institute of Parasitology, Biology Centre, Academy of Sciences of the Czech Republic, Branišovská 31, 370 05 České Budějovice, Czech Republic; 3 Department of Zoology, University of Otago, PO Box 56, Dunedin, New Zealand; 4 Marine Mammal Laboratory, National Patagonic Center, CONICET and University of Patagonia, Boulevard Brown 2915 (9120), Puerto Madryn, Chubut Argentina

**Keywords:** Anisakidae, Eealworms, *Pseudoterranova cattani*, Taxonomy, Ecology, *Cox*1, Marine fishes, Southwestern Atlantic

## Abstract

**Background:**

Third-stage larvae of the *Pseudoterranova decipiens* species complex (also known as sealworms) have been reported in at least 40 marine fish species belonging to 21 families and 10 orders along the South American coast. Sealworms are a cause for concern because they can infect humans who consume raw or undercooked fish. However, despite their economic and zoonotic importance, morphological and molecular characterization of species of *Pseudoterranova* in South America is still scarce.

**Methods:**

A total of 542 individual fish from 20 species from the Patagonian coast of Argentina were examined for sealworms. The body cavity, the muscles, internal organs, and the mesenteries were examined to detect nematodes. Sealworm larvae were removed from their capsules and fixed in 70% ethanol. For molecular identification, partial fragments of the mitochondrial cytochrome c oxidase subunit 1 gene (*cox*1) were amplified for 10 isolates from 4 fish species. Morphological and morphometric data of sealworms were also obtained.

**Results:**

A total of 635 larvae were collected from 12 fish species. The most infected fish was *Prionotus nudigula*, followed by *Percophis brasiliensis*, *Acanthistius patachonicus*, *Paralichthys isosceles*, and *Pseudopercis semifasciata*. Sequences obtained for the *cox*1 of sealworms from *A. patachonicus*, *P. isosceles*, *P. brasiliensis* and *P. nudigula* formed a reciprocally monophyletic lineage with published sequences of adult specimens of *Pseudoterranova cattani* from the South American sea lion *Otaria flavescens*, and distinct from the remaining 5 species of *Pseudoterranova.* A morphological description, including drawings and scanning electron microscopy photomicrographs of these larvae is provided. Sealworms collected from Argentinean fishes did not differ in their diagnostic traits from the previously described larvae of *P. cattani.* However a discriminant analysis suggests that specimens from *P. nudigula* were significantly larger than those from other fishes. Most of the sealworms were collected encapsulated from the muscles and, to a lesser degree, from the mesenteries and the liver.

**Conclusions:**

We provided the first molecular identification, morphological description and microhabitat characterization of sealworm larvae from the Argentinean Patagonian coast. We also reported the infection levels of sealworms on 20 fish species in order to elucidate the life cycle of these nematodes in this area.

## Background

Anisakid nematodes belonging to the *Pseudoterranova decipiens* species complex (also known as sealworms or codworms) mature and reproduce in the digestive tract of pinnipeds [1-3]. The sealworm larvae are much more disperse within the marine food webs and they propagate through complex feeding interactions ([4] and references therein). As far as it is known, the life cycle of species of *Pseudoterranova* also includes crustaceans as the first hosts, and fish as second hosts. The *P. decipiens* complex is composed of 6 sibling species, with 4 species occurring in the Northern Hemisphere, namely, *P. azarasi* (Yamaguti & Arima, 1942), *P. bulbosa* (Cobb, 1888), *P. decipiens* sensu stricto (s.s.) (Krabbe, 1868) and *P. krabbei* Paggi, Mattiucci, Gibson, Berland, Nascetti, Cianchi & Bullini, 2000; and 2 species in the Southern Hemisphere: *P. cattani* George-Nascimento & Urrutia, 2000 and *P. decipiens* E (sensu Bullini *et al.*[5]) see [3,5-9].

The third-stage larvae (L3) of sealworms have commonly been reported in marine teleosts worldwide [3,10-12]. Just along the South America coasts, sealworm larvae have been reported in at least 40 species of marine fish belonging to 21 families and 10 orders see (Additional file 1: Table S1). In this region, sealworm larvae infect the flesh of economically important fishes e.g. [13-15] and cause zoonotic diseases when humans consume raw or undercooked fish [16,17].

Despite the wide range of hosts infected with sealworms along the South American coast, morphological and molecular characterization of species of *Pseudoterranova* is still scarce. In the southeastern Pacific, Torres and González [18] provided the first biometrical and morphological data of the L3 of *Pseudoterranova* (=*Phocanema*) sp. from the liver of *Genypterus* sp. Later, Cattan and Carvajal [19] described adult specimens of *Pseudoterranova* (=*Phocanema*) *decipiens* sensu lato (s.l*.*) collected from the stomach of the South American sea lion *Otaria flavescens* (Shaw), hereinafter referred to as sea lion. George-Nascimento and Llanos [20] reported biometrical, morphological, and electrophoretic data from both L3 and adult specimens of *Pseudoterranova* sp. collected from marine fishes and sea lions, respectively, in the southeastern South Pacific. Subsequently, George-Nascimento and Urrutia [8] described *P. cattani* from sea lions, and identified the sealworm larvae reported by George-Nascimento and Llanos [20] as the L3 of this species. With regard to the southwestern Atlantic, Hernández-Orts *et al*. [21] recently confirmed the occurrence of fourth-stage larvae and adults of *P. cattani* in sea lions and in the South American fur seals *Arctocephalus australis* (Zimmerman) along the Patagonian coast of Argentina. Preliminary evidence also reports the occurrence of L3 of *P. cattani* in marine fishes caught off Argentina [22].

In this paper we provide, for the first time, molecular, morphological, and ecological data on the L3 of *Pseudoterranova* sp. in the Patagonian coast of Argentina based on an extensive parasitological survey on 20 fish species. We first carried out a molecular identification of larvae, followed by a morphological description of specimens, including examination by scanning microscopy. Second, we compared morphometric data of L3s collected from different fish species to investigate patterns of morphological variability. Finally, we report on infection levels among fish species and examine the distribution of larvae in fish, to provide a better understanding of the ecology of sealworms in the Patagonian coasts of Argentina.

## Methods

### Sample collection

**Figure 1:**
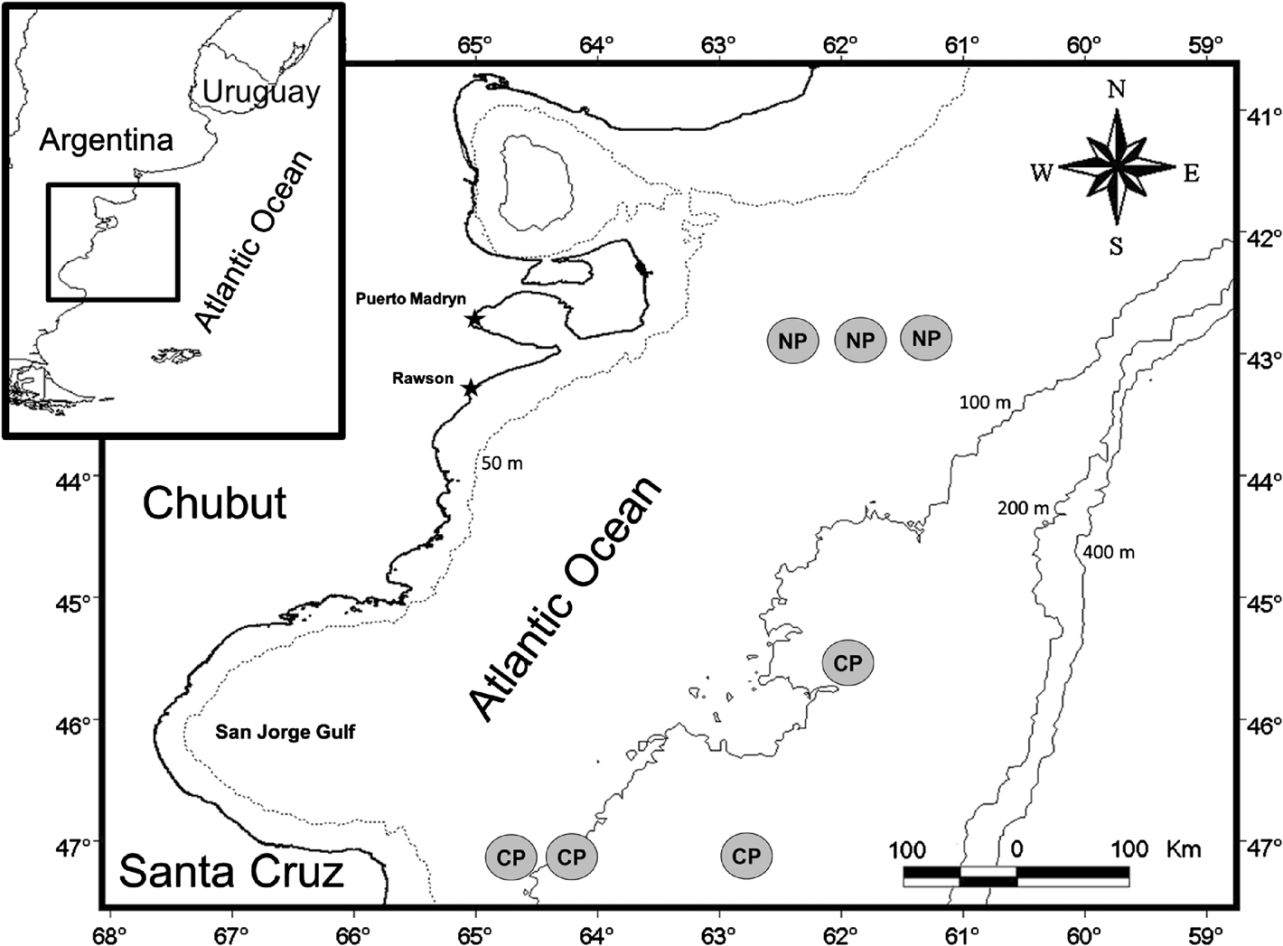
**Map of the Patagonian coast of Argentina showing the study area and the sampling sites (grey circles).***Abbreviations:* CP, central Patagonia; NP, North Patagonia.

A total of 542 individual fish from 20 species were examined for sealworm larvae (Table 1). Fishes were caught by commercial bottom trawling vessels during 2006–2007 along 2 areas of the Patagonian coast of Argentina: North (42°45′–42°59′S, 61°09′–62°58′W; depth range: 72–88 m) and central Patagonia (47°00′–47°19′S, 61°59′–64°25′W; depth range: 82–119 m) (Figure 1). Fishes were kept on ice on board and, after arrival to the laboratory, identified according to Menni *et al*. [23]. Fish scientific names were validated according to Froese and Pauly [24]. Specimens were then either examined fresh or frozen in plastic bags at –20°C for later examination. Fresh or thawed fishes were dissected, and internal organs were removed from the carcass. The body cavity was examined by naked eye, whereas the epaxial and hypaxial muscles regions, internal organs (liver, stomach, intestine and intestinal caeca, swim bladder, gonads) and mesenteries were pressed between Petri dishes and examined under a stereomicroscope (up to 40×) to detect encapsulated nematodes*.* Sealworm larvae were removed from their capsules, washed in saline and fixed in 70% ethanol. Three fish species have not been included in the study due to their low sampling size and absence of sealworms: the Atlantic pomfret *Brama brama* (Bonnaterre), the “rubio” *Helicolenus lahillei* Norman, and the Patagonian grenadier *Macruronus magellanicus* Lönnberg.

**Table 1:** **Biological data of the fish species examined for the presence of larvae of *Pseudoterranova *sp. from the Patagonian coast of Argentina**

**Host**	**n**	**Total length**
Gadiformes: Merlucciidae		
*Macruronus magellanicus* Lönnberg	3	56.7 ± 23.0 (40.1–83.0)
*Merluccius hubbsi* Marini	79	28.1 ± 4.2 (16.5–34.2)
Ophidiiformes: Ophidiidae		
*Genypterus blacodes* (Forster)	44	39.7 ± 9.4 (24.7–58.5)
*Raneya brasiliensis* (Kaup)	16	21.2 ± 1.4 (18.2–23.3)
Perciformes: Bramidae		
*Brama brama* (Bonnaterre)	2	60.5 ± 2.1 (59.0–62.0)
Perciformes: Bovichtidae		
*Cottoperca gobio* (Günther)	8	30.2 ± 9.3 (22.0–52.0)
Perciformes: Centrolophidae		
*Seriolella porosa* Guichenot	34	33.0 ± 5.6 (22.7–42.7)
Perciformes: Cheilodactylidae		
*Nemadactylus bergi* (Norman)	32	25.6 ± 5.5 (11.4–34.6)
Perciformes: Mullidae		
*Mullus argentinae* Hubbs & Marini	2	20.7 ± 0.4 (20.4–21.0)
Perciformes: Nototheniidae		
*Patagonotothen ramsayi* (Regan)	84	24.9 ± 3.5 (14.7–31.7)
Perciformes: Percophidae		
*Percophis brasiliensis* Quoy & Gaimard	8	45.3 ± 4.9 (37.1–51.8)
Perciformes: Pinguipedidae		
*Pseudopercis semifasciata* (Cuvier)	31	26.5 ± 2.7 (22.3–32.2)
Perciformes: Scombridae		
*Scomber japonicus* Houttuyn	13	42.7 ± 5.0 (32.5–48.0)
Perciformes: Serranidae		
*Acanthistius patachonicus* (Jenyns)	16	30.0 ± 2.6 (24.1–34.2)
Perciformes: Stromateidae		
*Stromateus brasiliensis* Fowler	73	27.5 ± 3.6 (13.7–36.4)
Pleuronectiformes: Paralichthyidae		
*Paralichthys isosceles* Jordan	15	27.2 ± 5.3 (17.9–34.4)
*Xystreurys rasile* (Jordan)	29	32.8 ± 5.8 (21.9–42.6)
Scorpaeniformes: Congiopodidae		
*Congiopodus peruvianus* (Cuvier)	15	23.9 ± 2.0 (21.0–28.0)
Scorpaeniformes: Sebastidae		
*Helicolenus lahillei* Norman	6	28.8 ± 2.6 (25.9–32.5)
Scorpaeniformes: Triglidae		
*Prionotus nudigula* Ginsburg	32	23.1 ± 2.8 (16.7–27.8)

Fish total length is given in centimetres as the mean ± standard deviation, followed by the range in parentheses.

### Molecular analysis

Total genomic DNA was isolated from the central part of the body of 20 specimens of *Pseudoterranova* sp. collected from 4 fish species, the Argentine seabass *Acanthistius patachonicus* (Jenyns) (n = 5), the flounder *Paralichthys isosceles* Jordan (n = 3), the Brazilian flathead *Percophis brasiliensis* Quoy & Gaimard (n = 5), and the red searobin *Prionotus nudigula* Ginsburg (n = 7). The specimens were fixed in 70% ethanol, and the anterior and posterior ends of each specimen were deposited as voucher specimens. DNA extractions consisted of placing individual isolates into 1.5 ml tubes in 300 μl of 5% chelex containing 0.1 mg/ml proteinase K, incubating at 60°C overnight, boiling at 90°C for 8 min and centrifuging at 15,000 g for 10 min. The mitochondrial cytochrome c oxidase subunit 1 gene (*cox*1) is a commonly used molecular marker for barcoding and prospecting species in numerous groups including some parasitic helminths [25,26]. We selected this marker because comparable *cox*1 sequences of *Pseudoterranova* spp. already exist in GenBank and, at the same time, it would allow us to rule out the possibility of cryptic species. We amplified partial *cox*1 sequences using primers JB3 (forward 5′-TTTTTTGGGCATCCTGAGGTTTAT-3′) and JB4 (reverse 5′-TAAAGAAAGAACATAATGAAAATG-3′) [27]. Polymerase chain reaction (PCR) amplifications were performed with 25 μl reactions containing 2.5 μl of extraction supernatant, 1X PCR buffer (16mM (NH_4_)_2_SO_4_, 67 mM Tris–HCl at pH 8.8), 2 mM MgCl_2_, 200 μM of each dNTP, 0.5 mM each primer, and 0.7 unit MyFi DNA polymerase (Bioline Ltd.). The following thermocycling profile was used for amplification: denaturation of DNA (95°C for 3 min); 35 cycles of amplification (94°C for 40 s, 50°C for 30 s and 72°C for 45 s); and 4 min extension hold at 72°C. PCR products were purified using PCR Product Pre-Sequencing Kit™ (Affymetrix/USB corporation). PCR primers were used for sequencing and PCR amplicons were cycle-sequenced from both strands using ABI BigDye™ Terminator v3.1 Ready Sequencing Kit, ethanol-precipitated, and run on an ABI 3730xl automated sequencer. Contiguous sequences were assembled and edited using MEGA 5.0 [28].

In order to examine the affinity of our isolates with other species of *Pseudoterranova* the newly obtained sequences for *cox*1 were aligned together with 15 sequences available from GenBank obtained by Cao *et al*. [29] (GenBank accession numbers for these sequences were not provided in their study): *P. azarasi, P. bulbosa*, *P. cattani*, *P. decipiens* (s.l*.*), *P. decipiens* (s.s.)*, P. decipiens* and *P. krabbei* (see Table 2 for details). Newly-generated and published *cox*1 sequences for *Pseudoterranova* spp. were aligned using MUSCLE implemented in MEGA with default parameter values, with references to the amino acid translation, using the invertebrate mitochondrial code [28]. Sequences of two species of *Contracaecum* Railliet & Henry, 1913 were used as outgroups (Table 2). Phylogenetic trees were built under Bayesian Inference (BI) and Maximum Likelihood (ML) criteria. BI analysis was performed in MrBayes 3.2 [30] using Markov chain Monte Carlo searches on two simultaneous runs of four chains during 10^7^ generations, sampling trees every 10^3^ generations. The evolutionary substitution model GTR (general time-reversible model) was applied and the parameter gamma was allowed to accommodate among-site rate variation. The first 10^3^ trees sampled were discarded as ‘burn-in’, as determined by stationarity of lnL assessed using Tracer v. 1.4 [31], and a consensus topology and nodal support estimated as posterior probability values [32] were calculated from the remaining trees. ML analysis was conducted using the program PhyML3.0 [33]. All model parameters and bootstrap nodal support values (1000 repetitions) were estimated. Mean genetic distances (raw p-distance) between and within species, and standard deviation (S.D.) estimates were calculated on 500 bootstrap replicates with MEGA v5.

**Table 2:** **Sequence information of species of *pseudoterranova *and *contracaecum *used in the molecular analysis**

**Species**	**Host name**	**Developmental stage**	**Locality**	**Isolates code**	**GenBank cox 1**
*P. azarasi*	*Eumetopias jubatus*	Adult	Iwanai, Japan		AJ891139
					AJ891140
*P. bulbosa*	*Erignathus barbatus*	Adult	Newfoundland, Canada		AJ891141
					AJ891142
*P. cattani*	*Otaria flavescens*	Adult	Concepcion, Chile		AJ891143
	(syn. *Otaria byronia*)				AJ891144
	*Acanthistius patachonicus*	Larva	Patagonia, Argentina	Ap1	KF545942**
				Ap2	KF545943**
				Ap3	KF545944**
	*Paralichthys isosceles*			Pi1	KF545948**
	*Percophis brasiliensis*			Pb1	KF545945**
				Pb2	KF545946**
				Pb3	KF545947**
	*Prionotus nudigula*			Pn1	KF545949**
				Pn2	KF545950**
				Pn3	KF545951**
*P. decipiens* (s.s.)*	*Phoca vitulina*	Adult	Newfoundland, Canada		AJ891145
*P. decipiens* (s.l.)*	*Osmerus eperlanus*	Larva	Elbe estuary, Germany		AJ891150
	*Chaenocephalus aceratus*	Larva	Elbe estuary, Germany		AJ891146
					AJ891147
					AJ891148
					AJ891149
*P. krabbei*	*Halichoerus grypus*	Adult	Froya Island, Norway		AJ891151
					AJ891152
					AJ891153
*C. osculatum*†	–	–	Australia/Antarctic		AJ405315
*C. rudolphii* C†	–	–	Florida, US		FJ866816

*Species epithets for *P. decipiens* isolates according to Cao *et al*. [29].

**Sequences obtained in the present study.

†Species included as outgroup.

### Morphological analyses

All the larvae (n = 635) were examined under stereomicroscope, bright field microscope, or differential interference contrast microscope. Some of them were cleared in glycerin or lactophenol to ease their identification. The measurements of the larvae were taken from drawings made with the aid of a drawing tube and are expressed in millimetres. For the morphological description of sealworm larvae, measurements are presented as the mean followed by S.D., with the range and sample size in parentheses. Voucher specimens are deposited in the Natural History Museum, London, UK (accession numbers: 2012.5.15.144-172), and the Helminthological Collection of the Institute of Parasitology (IPCAS), Biology Centre ASCR, České Budějovice, Czech Republic (accession numbers: N-1013).

Also, some larvae from *P. nudigula* were studied externally and internally with scanning electron microscopy (SEM). Three larvae fixed in 70% ethanol were dissected and the body wall partially removed to examine the morphology of the anterior part of the digestive tract. All the specimens were dehydrated through an ethanol series, critical point dried, and coated with a gold-palladium alloy to a thickness of 250 nm. Specimens were examined with a Hitachi 4100 FE scanning electron microscope, operating at 20 kV, from the Central Service for the Support to Experimental Research of the University of Valencia.

Morphometric variation of sealworm larvae from different fish species was examined through a discriminant analysis, based on canonical distances. Morphometric data were obtained from 8 specimens collected from *A. patachonicus*, 27 from *P. brasiliensis*, and 80 from *P. nudigula*. Only specimens in good condition were selected for this analysis. Multivariate statistical analysis were performed on 8 metrical variables: body length, body width, distance from anterior end to nerve-ring, distance from distal end of excretory gland to posterior end, muscular oesophagus length, glandular ventriculus length, intestinal caecum length and tail length. The canonical discriminant functions were calculated using all variables simultaneously. Statistical analysis was carried out with SPSS v19. Statistical significance was set at p < 0.05.

### Ecological analyses

Ecological terms follow Bush *et al*. [34] and Rózsa *et al*. [35]. The prevalence, mean abundance and mean intensity are followed by the 95% confidence intervals (C.I.) in parentheses. The 95% C.I. for prevalence was set with Sterne’s exact method [36], whereas the 95% C.I.s for the mean abundance and mean intensity were estimated with 20,000 bootstrap replications using the statistical software Quantitative Parasitology 3.0 [37].

A preliminary analysis indicated no significant differences in the abundance of sealworm larvae between species of fish collected in different sampling sites (Mann–Whitney test, p > 0.05, Figure 1). Therefore, infection parameters were calculated for and statistical analyses were carried out with the pooled data.

Differences in the intensity of L3 of *Pseudoterranova* between species of fish were investigated with the Kruskal-Wallis test followed by post hoc comparisons between fish species [38]. For this analysis we considered only fish species with a sample size > 5 individuals.

## Results

### Molecular identification

**Figure 2:**
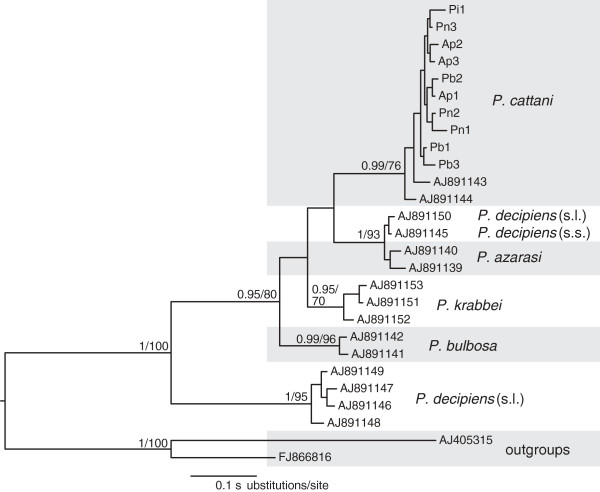
**Phylogram derived from Bayesian analysis of *cox*1 sequences (364 bp) of six *Pseudoterranova *spp.** Sequences of *Contracaecum rudolphii* and *C. osculatum* were used as outgroups. Posterior probabilities (PP) followed by bootstrap support (BS) values are indicated at the nodes (PP < 0.95 and BS < 60 were omitted). Newly sequenced isolates are indicated with abbreviation of its fish host species followed by a number: Ap, *Acanthistius patachonicus*; Pi, *Paralichthys isosceles*; Pb, *Percophis brasiliensis* and Pn, *Prionotus nudigula*. Species epithets for *P. decipiens* isolates according to Cao *et al*. [29].

A total of 10 partial *cox*1 sequences were generated for sealworm larvae from Argentinean fishes (3 from *A. patachonicus*, 1 from *P. isosceles*, 3 from *P. brasiliensis*, and 3 from *P. nudigula*; Table 2). These sequences were aligned with the available sequences for *cox*1 of *Pseudoterranova* spp. (Table 2), resulting in a dataset of 25 sequences, which was comprised of 364 nt positions after trimming the ends to match the shortest aligned sequences. The newly generated sequences formed a strongly supported clade together with representative sequences of adult specimens of *P. cattani* from sea lion of Chile, and distinct from the remaining 4 species of *Pseudoterranova* included in the analysis (Figure 2). Mean genetic divergence among species ranged between 5.1–11.4%. Intraspecific mean genetic divergence ranged between 1.1–1.4%. For isolates of *P. cattani*, including the newly generated sequences from Argentinean fishes, the intraspecific mean genetic divergence was 1.2 ± 0.3%. Overall, molecular results suggest that sealworm larvae from these hosts belong to *P. cattani*.

One isolate labelled as *P. decipiens* (s.s.) and another as *P. decipiens* (s.l*.*) (see Table one in Cao *et al*. [29] but not specified in the GenBank records), clustered as sister to two isolates of *P. azarasi*, being distant from the clade formed by 4 other *P. decipiens* (s.l.) isolate sequences (Figure 2). The low mean divergence between *P. decipiens* (s.s.) isolate AJ891145 and *P. decipiens* (s.l.) isolate AJ891150 and isolates of *P. azarasi* (1.3 ± 0.4%) falls within the range of intraspecific divergence for the group, suggesting that these four isolates may be conspecific.

### Morphological description of *P. cattani* from *P. nudigula*

#### Third-stage larvae

**Figure 3:**
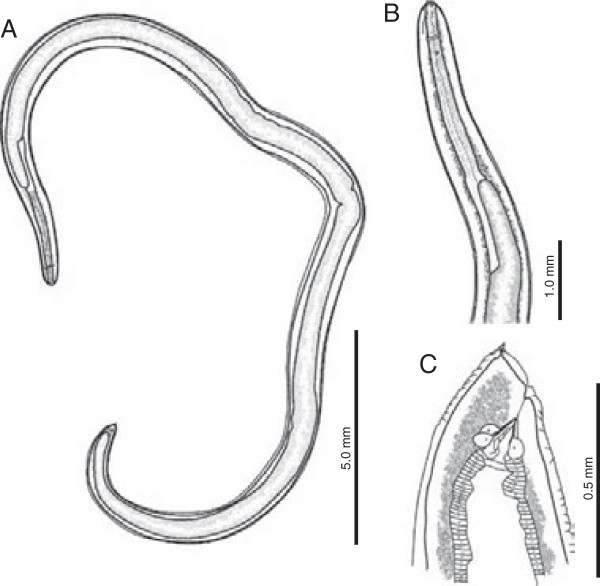
**Third-stage larvae of *Pseudoterranova cattani *collected from *Prionotus nudigula. *A**. Whole worm, lateral view. **B**. Anterior end, lateral view. **C**. Posterior end, lateral view.

**Figure 4:**
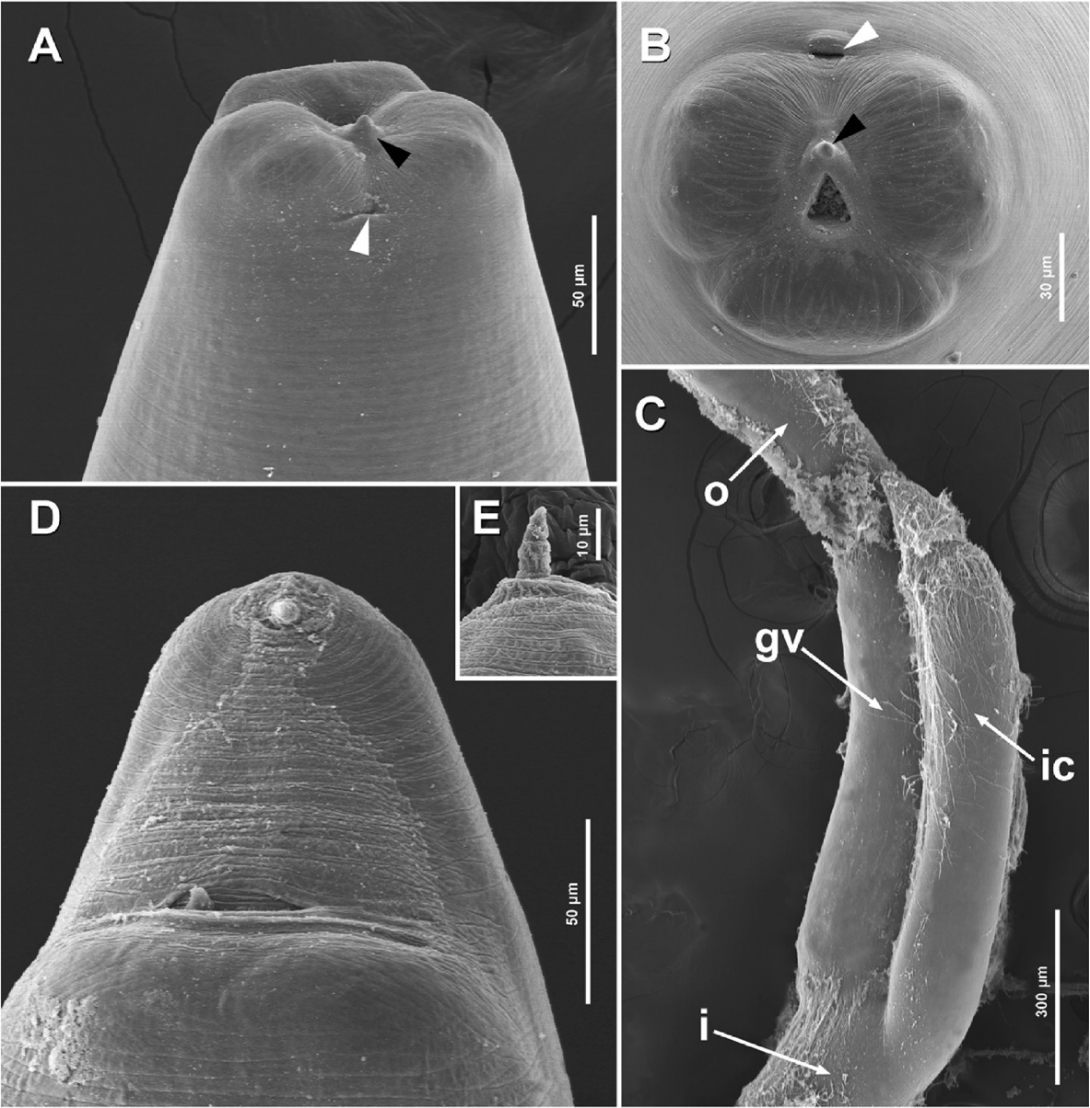
**Scanning electron micrographs of the third-stage larvae of *Pseudoterranova cattani *collected from *Prionotus nudigula. *A**. Anterior end, ventral view. **B**. Anterior end, apical view. **C**. Detail of the area of the glandular ventriculus and intestinal caecum of a dissected worm, lateral view. **D**. Posterior end, ventral view. **E**. Mucron, lateral view. Black arrowheads point to the boring tooth and white arrowheads point to the excretory pore. *Abbreviations*: i, intestine; ic, intestinal caecum; gv, glandular ventriculus; o, oesophagus.

Description based on 80 third-stage larvae examined by light microscopy and 7 specimens by SEM. Body yellowish to reddish, medium-sized, elongate, 31.1 ± 3.6 (23.8–43.2, n = 80) long by 0.9 ± 0.1 (0.7–1.4, n = 80) wide (Figure 3A), with transverse striations along the whole body (Figures 4A, B and D). Anterior end rounded. Cuticle covering the triangular mouth aperture (Figure 4B), with 3 lips (2 ventro-lateral and 1 dorsal) of approximately equal size (Figure 4A and B). Each lip with a pair of soft swellings of the cuticle at level of papillae. Boring tooth antero-ventral, between the ventro-lateral lips (Figure 4A and B). Excretory pore opening ventrally (Figures 4A and B), below boring tooth. Nerve-ring at 0.4 ± 0.1 (0.2–0.6, n = 80) from anterior body end (Figures 3A and B). Deirids lateral, posterior to nerve-ring, about 0.7 ± 0.1 (0.5–1.0, n = 16) from anterior body end (Figure 3B). Muscular oesophagus 2.0 ± 0.2 (1.4–2.4, n = 80) long (Figures 3A and B). Oesophagus/body length ratio 0.1 ± 0.01 (0.04–0.1, n = 80). Glandular ventriculus 1.2 ± 0.2 (0.7–1.5, n = 80) long (Figures 3A, B and C). Glandular ventriculus/body length ratio 0.04 ± 0.01 (0.02–0.1, n = 80). Intestinal caecum 1.1 ± 0.2 (0.4–1.7, n = 80) long (Figures 3A, B and C). Intestinal caecum/body length ratio 0.04 ± 0.01 (0.02–0.5, n = 80). Intestinal caecum shorter (n = 39), equal (n = 18) or slightly longer (n = 23) than glandular ventriculus. Intestinal caecum/glandular ventriculus length ratio 1.0 ± 0.1 (0.6–1.3, n = 80). Rectum surrounded by three rectal glands, one ventral and two dorsal (Figure 3C). Tail short, conical, pointed, 0.2 ± 0.03 (0.1–0.2, n = 80) long (mucron not included) (Figures 3C and 4D). Distance from distal end of excretory gland to posterior body end 0.2 ± 0.1 (0.1–0.4, n = 79). Mucron 0.02 ± 0.00 (0.01–0.04, n = 71) long (Figures 3C and 4E).

#### Taxonomic summary

*Fish host:* Red searobin *Prionotus nudigula* Ginsburg (Scorpaeniformes: Triglidae).

*Locality*: North Patagonia, Chubut Province, Argentina (42°45′–42°59′S, 61°09′–62°58′W).

*Site in the host*: liver, mesenteries and muscles.

*Infection parameters*: see Table 3.

**Table 3:** Occurrence of sealworm larvae in 12 species of marine fish from Patagonia, Argentina

**Host**	**n**	**Microhabitat**	**Prevalence (%)**	**Mean abundance**	**Mean intensity**	**Range**
		**(n sealworms collected)**	**(95% C.I.)**	**(95% C.I.)**	**(95% C.I.)**	
*A. patachonicus* (DB)*	16	Li (1), Me (2), Mu (11)	25.0 (9.0–50.0)	0.9 (0.2–2.3)	3.5 (1.5–5.5)	1–7
*C. peruvianus* (DB)	15	–	–	–	–	–
*C. gobio* (DB)	8	Mu (4)	12.5	0.5	4.0	4
*G. blacodes* (DB)	44	Li (2)	2.3 (0.1–12.1)	0.1 (0.0–0.1)	2.0	2
*M. hubbsi* (DP)	79	Me (2), Mu (1)	3.8 (1.1–10.6)	0.0 (0.0–0.1)	1.0	1
*M. argentinae* (DB)	2	Me (1), Mu (1)	100	1.0	1.0	1
*N. bergi* (DB)	32	Me (1)	3.1 (0.1–16.6)	0.0 (0.0–0.1)	1.0	1
*P. ramsayi* (DB)	84	–	–	–	–	–
*P. isosceles* (B)*	15	Me (2), Mu (9)	26.7 (9.7–53.4)	0.7 (0.1–2.3)	2.8 (1.0–5.5)	1-7
*P. brasiliensis* (DB)*	8	Mu (67)	25.0	8.4	33.5	29–38
*P. nudigula* (B)*	32	Li (1), Me (22), Mu (495)	100 (89.5–100)	16.2 (12.5–20.9)	16.2 (12.5–20.9)	1–50
*P. semifasciata* (DB)	31	Li (1), Mu (10)	25.8 (12.6–43.4)	0.4 (0.1–0.6)	1.4 (1.0–1.6)	1–2
*R. brasiliensis* (B)	16	–	–	–	–	–
*S. japonicus* (P)	13	Me (1)	7.7	0.1	1.0	1
*S. porosa* (DP)	34	–	–	–	–	–
*S. brasiliensis* (DP)	73	–	–	–	–	–
*X. rasile* (B)	29	Mu (1)	3.4 (0.2-16.8)	0.0 (0.0-0.1)	1.0	1

The ecological group for each species is shown in parentheses after the host name. Ecological groups mainly assigned following Koen-Alonso *et al*. [62] and Romero *et al*. [63]. The 95% C.I. were only estimated for fish species with n ≥ 15. *Abbreviations: B* benthic, *DB* demersal–benthic, *DP* demersal–pelagic, *Li* liver, *Me* mesenteries, *Mu* muscles, *P* pelagic.

*Sealworms molecularly identified as *Pseudoterranova cattani* George-Nascimento & Urrutia, 2000 (n = 3; except *P. isosceles*, n = 1).

### Morphometric comparison of larvae among fish species

Morphometric data obtained from L3 of *P. cattani* from *A. patachonicus, P. brasiliensis* and *P. nudigula* are presented in Table 4. Overall morphometric differences of larvae from the 3 fish species from the Patagonian coast of Argentina were highly significant. Univariate tests indicated that 6 out of the 8 variables significantly differ among groups, i.e. body length (Wilks’ λ = 0.650, F_(2,110)_ = 29.645, p < 0.001), body width (Wilks’ λ = 0.663, F_(2,110)_ = 0.27.932, p < 0.001), distance from anterior end to nerve-ring (Wilks’ λ = 0.717, F_(2,110)_ = 21.657, p < 0.001), muscular oesophagus length (Wilks’ λ = 0.668, F_(2,110)_ = 27.306, p < 0.001), glandular ventriculus length (Wilks’ λ = 0.699, F_(2,110)_ = 23.646, p < 0.001), and intestinal caecum length (Wilks’ λ = 0.755, F_(2,110)_ = 17.894, p < 0.001).

**Table 4:** **Morphometric data of third-stage larvae of *Pseudoterranova cattani *in seven fish species from South America**

**Reference**	**George-Nascimento &Llanos [**[20]]*				**Present study**		
Hosts	*Cilus gilberti*	*Genypterus maculatus*	*Merluccius gayi gayi*	*Paralichthys microps*	*Acanthistiu patachonicus*	*Percophis brasiliensis*	*Prionotus nudigula*
Locality	Talcahuano,	Talcahuano,	Talcahuano,	Talcahuano,	Chubut,	Chubut,	Chubut,
	Chile	Chile	Chile	Chile	Argentina	Argentina	Argentina
n	15	15	15	15	8	27	80
BL (mm)	30.1 ± 3.6	30.6 ± 5.8	28.5 ± 4.3	29.0 ± 5.8	18.3 ± 5.6 (8.4–23.8)	28.2 ± 5.1 (17.1–40.5)	31.1 ± 3.6 (23.8–43.2)
BW (mm)	0.7 ± 0.1	0.7 ± 0.1	0.8 ± 0.1	0.7 ± 0.1	0.6 ± 0.2 (0.5–0.8)	0.8 ± 0.1 (0.5–0.9)	0.9 ± 0.1 (0.7–1.4)
OL (mm)	1.7 ±0.2	1.7 ± 0.2	1.6 ± 0.3	1.6 ± 0.2	1.5 ± 0.3 (1.0–1.8)	1.8 ± 1.2 (1.4–2.1)	2.0 ± 0.2 (1.4–2.4)
GVL (mm)	1.0 ± 0.1	0.9 ± 0.1	0.8 ± 0.3	1.0 ± 0.1	0.8 ± 0.2 (0.5 – 1.0)	1.0 ± 0.1 (0.7–1.3)	1.2 ± 0.2 (0.7–1.5)
ICL (mm)	0.8 ± 0.1	0.7 ± 0.2	0.7 ± 0.2	0.8 ± 0.2	0.7 ± 0.1 (0.5–0.8)	1.0 ± 0.2 (0.6–1.7)	1.1 ± 0.2 (0.4–1.7)
TL (mm)	0.1 ± 0.0	0.1 ± 0.0	0.1 ± 0.0	0.1 ± 0.0	0.1 ± 0.0 (0.1–0.2)	0.1 ± 0.0 (0.1–0.2)	0.2 ± 0.0 (0.1–0.2)
BL/BW	41.3 ± 6.9	42.0 ± 8.4	35.4 ± 6.7	42.4 ± 6.7	28.6 ± 5.4 (16.8–34.0)	37.8 ± 5.1 (22.1–50.6)	34.3 ± 5.3 (22.8–47.9)
BL/OL	17.5 ± 2.3	17.8 ± 2.9	18.4 ± 4.1	17.9 ± 3.3	11.8 ± 2.1 (8.4–14.9)	15.7 ± 2.2 (10.9–20.8)	15.5 ±2.3 (11.5–22.4)
BL/GVL	30.6 ± 4.7	34.3 ± 5.2	37.5 ± 11.6	29.9 ± 5.1	25.7 ± 8.5 (15.4–36.8)	27.4 ± 4.5 (18.4–36.8)	27.3 ± 4.8 (20.5–40.3)
BL/ICL	38.0 ± 6.5	44.0 ± 8.6	42.1 ± 15.6	39.3 ± 9.8	28.8 ± 9.5 (18.0–45.2)	28.8 ± 4.8 (18.3–37.5)	29.0 ± 6.8 (20.2–59.8)
GVL/ICL	1.3 ± 0.3	1.3 ± 0.3	1.2 ± 0.5	1.3 ± 0.3	1.2 ± 0.2 (0.9–1.4)	1.1 ± 0.1 (0.8–1.3)	1.1 ± 0.2 (0.8–1.6)
BL/TL	277.1 ± 58.7	289.9 ± 46.3	235.3 ± 73.3	267.9 ± 127.0	133.2 ± 45.6 (76.4–210.0)	199.3 ± 45.4 (134.9–334.7)	205.6 ± 40.4 (114.4–305.0)

*Abbreviations: BL* body length, *BW* body width, *GVL* glandular ventriculus length, *ICL* intestinal caecum length, *OL* oesophagus length, *TL* tail length.

*Later classified as *P. cattani* by George-Nascimento & Urrutia [8].

**Figure 5:**
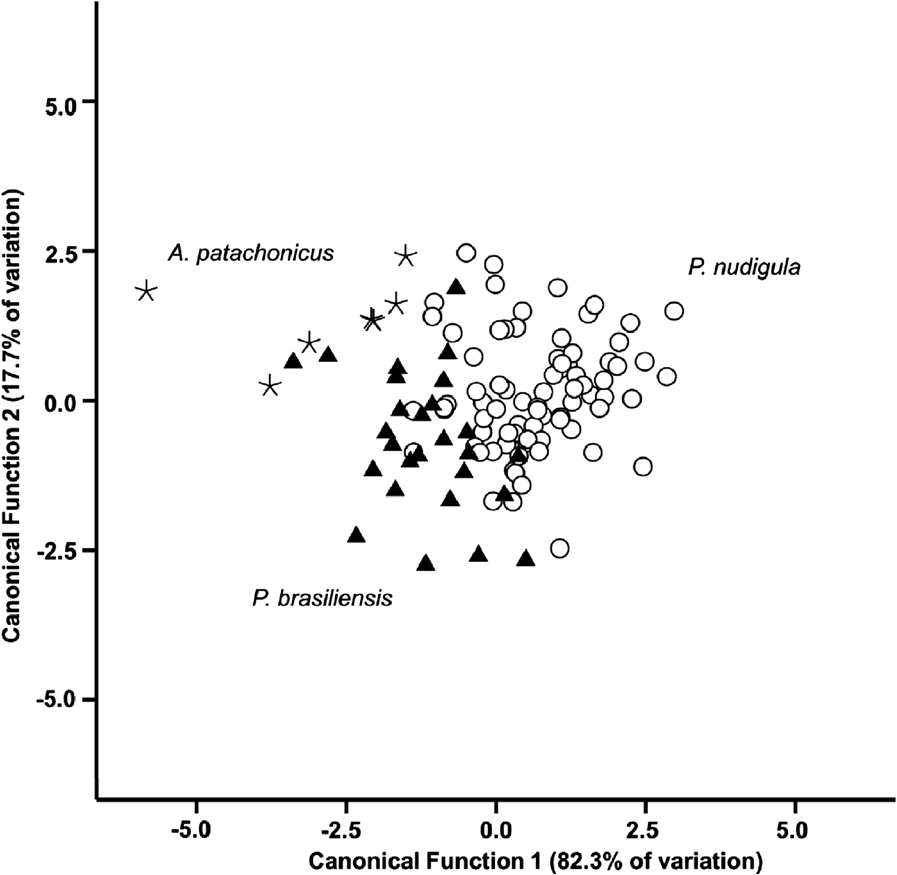
**Plot of the 9 metrical variables measured from third-stage larvae of *Pseudoterranova cattani *collected from *Prionotus nudigula *(n = 80), *Acanthistius patachonicus *(n = 8) and *Percophis brasiliensis *(n = 27), against the first and second canonical discriminant functions.**

Functions 1 and 2 of the discriminant analysis accounted for 82.3% (eigenvalue = 1.168) and 17.7% (eigenvalue = 0.252) respectively (Figure 5). The variables showing high absolute values of standardized coefficients along the first function were the glandular ventriculus length (0.44), the distance from anterior end to nerve-ring (0.41) and the width of the body (0.35). Although the specimens of *P. cattani* from *P. nudigula* mostly overlap with those from *P. brasiliensis* in the first function (Figure 5), these variables tend to be relatively larger to those of sealworm larvae from other fishes. For the second function, the variables showing high absolute values of standardized coefficients were the length of the body length (-0.75), distance from anterior end to nerve-ring (0.45) and the width of the body (0.43). These suggest that sealworms from *P. brasiliensis* will tend to show relatively lower body length than those of sealworms from the other two fishes.

A comparison of the morphometric data from L3 of *P. cattani* of the Patagonian coast of Argentina and from other fish species in Chile is shown in Table 4. Morphometric measurements obtained from sealworm larvae from *P. nudigula* and *P. brasiliensis* overlap with those reported by George-Nascimento and Llanos [20] from L3 of *P. cattani* infecting 4 marine fish species from the southeastern Pacific coast of Chile (Table 4). No clear differences were detected between sealworm larvae from both geographical areas, except that some specimens collected from *A. patachonicus* are apparently smaller.

### Ecological patterns

A total of 635 third-stage sealworm larvae were collected from 12 marine fish species from the Patagonian coast in Argentina (Table 3). Five species of fish, i.e. *Cottoperca gobio* (Günther), *Nemadactylus bergi* (Norman), *Mullus argentinae* Hubbs & Marini, *Percophis brasiliensis* and *Prionotus nudigula* represent new host records for sealworms. The smallest infected fish with sealworm larvae was a *P. nudigula* 16.7 cm long (intensity, 1), whereas the largest was a *G. blacodes*, 58.0 cm long (intensity, 2). A specimen of *P. nudigula* of 20.7 cm long presented the highest infection (intensity, 50).

A comparison of sealworm abundance between fish species revealed highly significant differences (Kruskal-Wallis test, *χ*^2^ = 336.141, 16 d.f., p < 0.001); the post hoc test (p < 0.05) indicated that only *P. nudigula* harboured significantly more sealworm larvae than any other fish species. The abundance of sealworm larvae was weakly, but significantly correlated with body length only in *P. nudigula* (Spearman rank correlation: r_s_ = 0.324, n = 32, one-tailed p = 0.035).

In *P. nudigula*, most of the L3 of *P. cattani* were found encapsulated in the epaxial muscles (number of sealworms collected, 315; mean abundance [95% C.I.], 9.8 [7.3–13.1]). Sealworms were also abundant in the hypaxial muscles (180; 5.6 [4.3–7.2]) and, to a lesser extent, in the mesenteries (22; 0.7 [0.4–1.1]) and the liver (1; 0.03 [0.0–0.1]). Differences in the number of larvae between microhabitats were all highly significant (Friedman test, *χ*^2^ = 71.544, 3 d.f., p < 0.001; in all post hoc comparisons, p < 0.005). In other species too, sealworm larvae were generally collected from the muscles, and to a lesser extent, the mesenteries and liver (Table 3).

## Discussion

### Identification and morphometric variability of sealworm larvae from Patagonia, Argentina

Morphological characters for species identification in anisakid nematodes are few and mostly used to differentiate adult specimens. Molecular genetic markers are necessary to reliably identify species based on larvae as they are morphologically indistinguishable [3,39]. At present, most of the studies identifying sealworm larvae from fish using molecular tools have been performed in the Northern Hemisphere e.g. [2,7,39-41], as these nematodes are responsible for great economic losses to the fishing industry [42]. In contrast, in the Southern Hemisphere, and especially along the South American coasts, accurate species-level identification of sealworm larvae infecting fish is still scarce. Currently *P. cattani* is the only species of sealworm that has been identified using molecular tools in South America [3,43]. This species has been reported in 4 species of marine fishes in the southeastern Pacific coast of Chile, i.e. the corvina drum *Cilus gilberti* (Abbott), the black cusk-eel *Genypterus maculatus* (Tschudi), the South Pacific hake *Merluccius gayi gayi* (Guichenot) and the flatfish *Paralichthys microps* (Günther) [20,44]. Preliminary genetic studies indicated that larvae of *P. cattani* also occur in fishes from in the southwestern Atlantic coast of Argentina, including *A. patachonicus,* the flatfish *Paralichthys patagonicus* Jordan, and the Argentinian sandperch *Pseudopercis semifasciata* (Cuvier) [22]. Overall, geographical data of infection of *P. cattani* in fishes are congruent with the reports of adults in Chile [8], and the Patagonian coast of Argentina [21].

In the present study, the molecular data obtained from the 10 sealworm specimens collected from 4 fish species indicate that these larvae can be identified as *P. cattani*. The newly generated sequences formed a strongly supported and very consistent clade together with the available isolates of adults of *P. cattani* from sea lions. Unexpectedly, some isolates of other *Pseudoterranova* species from previous studies formed apparently incongruent groupings (e.g. the close clustering of the isolates from *P. decipiens* (s.l.), *P. decipiens* (s.s.) and *P. azarasi*, pointing to conspecificity). In view of these results, a comprehensive and detailed morphological and molecular study is necessary in order to properly establish the intra- and interspecific relations of the species of *Pseudoterranova*.

Larvae of *Pseudoterranova* found in other fish species of this study probably belong to a single species, i.e. *P. cattani,* as diagnostic traits are very similar. However, L3 of *P. cattani* from *P. brasiliensis* and specially those collected from *A. patachonicus* were significantly smaller (including some internal organs) than those from *P. nudigula*. These morphometric differences could be related to three factors: *i*) the small number of specimens studied (8 sealworms from *A. patachonicus* vs. 80 from *P. nudigula*); *ii*) the degree of development of the sealworm larvae, i.e. recently recruited sealworm larvae from the invertebrate host are smaller [45]; and *iii*) different fish species could have a significant effect on the morphometric values of sealworm larvae. In this respect, George Nascimento and Llanos [20] reported small, but significant morphometric variations in L3 of *P. cattani* collected from different fish species from the Chilean coast. Moreover, experimental evidence revealed that growth rate, and therefore the morphometric variables, of sealworm larvae, differ with host species [45]. Thereafter, morphometric variables must be considered with caution when differentiating sealworm larvae species infecting different fish hosts from close localities.

Finally, although SEM is commonly used to study the external traits of nematodes (see for example [46]), we would like to stress the possibilities of studying dissected anisakids and other nematodes in general using this technique. These SEM micrographs provide novel perspective on morphology and arrangement of the internal organs (e.g. the oesophagus and proximal intestine). Additionally, the observation of the internal organs with higher magnification could provide new diagnostic traits for the taxonomy of other nematode species.

### Ecology of sealworms from the Patagonian coasts of Argentina

Knowledge on the life cycle of the species belonging to the *P. decipiens* complex is scarce, and has mainly been obtained from natural and experimental evidences from sealworms and their hosts from the Northern Hemisphere. The complete life cycle of *P. decipiens* (s.s.) was summarized by McClelland [42], and includes mainly copepods, macro invertebrates (e.g. polychaetes and decapods), fishes and several species of pinnipeds. Although the host species involved in the life cycle may differ among sealworm species, we followed McClelland [42] to elucidate some parts of the life cycle of sealworms inhabiting the Patagonian coast.

To our knowledge, the identity of the invertebrate hosts for sealworms is unknown along the southwestern Atlantic coasts. However, in this area, sealworms could possibly infect a wide range of invertebrates hosts (e.g. copepods, mysids, isopods, decapods, etc.), like it has been reported in other species of *Pseudoterranova* from the Northern Hemisphere [47]. In the present study, the most infected fish, *P. nudigula* (see Table 3), feeds on small benthic invertebrates, mainly crustaceans [48]. Therefore, characterization of the food habits and prey of this fish from the Patagonian coast, could help to elucidate the specific identity of the invertebrate hosts for *P. cattani* in this area.

According to our results, in the Patagonia coast *P. nudigula* appears to be the primary fish host for sealworm larvae. Primary fish hosts are generally benthic consumers, which acquire the parasite directly from invertebrate hosts and are essential in the temporal and spatial dispersion of the larvae [42]. In this area, other sympatric benthic fishes, feeding on invertebrates, i.e. the banded cusk eel *Raneya brasiliensis* (Kaup) and the flatfishes *P. isosceles* and *Xystreurys rasile* (Jordan), lack or have been reported with low intensities of sealworm larvae compared with those of *P. nudigula* ([49]; see Table 3). Differences in the levels of sealworm infection on sympatric benthic fish are not surprising. Martel and McClelland [50] reported significant differences in the abundance of sealworm larvae in three sympatric flatfish species from Canada, which seems to be related to their food habits. Therefore, this could also be the main factor promoting differences in the infection levels of sealworms in benthic fishes from the Patagonian coast.

Our results also suggest that *P. brasiliensis* could be considered as secondary fish host in the Patagonian coast due to the high intensity of sealworm larvae encapsulated in the muscles. Secondary fish hosts of sealworms are commonly large demersal fish, which acquire the parasites by preying on smaller fish [42]. In other parasitological studies of large demersal ichthyophagous fishes from Patagonia, Timi and Lanfranchi [51] reported high prevalence but low intensity of sealworm larvae in specimens of *P. semifasciata* (> 60 cm TL) caught offshore from Península Valdés; while in the pink cusk-eel *Genypterus blacodes* (Forster) (syn. *G. brasiliensis* according to Froese and Pauly [24]) (> 40 cm TL) and the Brazilian sandperch *Pinguipes brasilianus* Cuvier (> 35 cm TL), sealworms reached the status of component species (prevalence > 10%), although the mean intensities are considerably low (< 2) [52,53]. Interestingly, the economically most important fish caught in the Patagonian waters, the Argentine hake *Merluccius hubbsi* Marini also presented low prevalences of sealworms (< 10%) in large specimens (> 57 cm TL) [14]. On the other hand, sealworms have also been reported in demersal cephalopods. Low prevalences of a L3 resembling *Pseudoterranova* sp. were reported from the Argentine shortfin squid *Illex argentinus* (Castellanos) from northern Patagonia [54]. Nevertheless, further parasitological surveys of different species of cephalopods are necessary in order to characterize the relative importance of these hosts in the biology and life cycle of sealworms.

Regarding pelagic fishes from the Patagonian coast, sealworm larvae were not recorded infecting the Argentine anchovy, *Engraulis anchoita* Hubbs & Marini [55], whereas low prevalences and intensities have been reported in the silversides *Odontesthes smitti* (Lahille) and *Odontesthes nigricans* (Richardson) [56,57], and in the chub mackerel *Scomber japonicus* Houttuyn (see Table 3). The mild infection of sealworms in pelagic fishes could be related with the benthic early stages in the life cycle of these nematodes.

At present, 3 potential definitive hosts of sealworms inhabit the Patagonian coast of Argentina: 2 otariids, sea lion and the South American fur seal; and one phocid, the southern elephant seal *Mirounga leonina* (L.) [58-61]. In this area, larvae and adults of *P. cattani* have been reported from the intestine of both species of otariids, [21]; while in elephant seals, a single adult specimen of *Pseudoterranova* sp. was collected from the intestine of a young female stranded in northern Patagonia [Hernández-Orts *et al*., unpublished data]. Although the 3 species of pinnipeds seem to be suitable definitive hosts for sealworms, the relative importance of these sympatric pinnipeds in the population dynamics of sealworms in this area is uncertain, as currently the abundance, morphology and fecundity of sealworms in these hosts are unknown.

In the Patagonian coast, pinnipeds will be infected with sealworm through the consumption of infected invertebrates, cephalopods or fishes. However, for most of the pinnipeds species inhabiting this area food habits are still unknown, making it difficult to study the transmission strategies of sealworm larvae. At present, only the food habits of sea lion have been characterized in detail from the Patagonian coast of Argentina [62,63]. According to these studies, sea lions feed mainly on *M. hubbsi*, *R. brasiliensis*, *E. anchoita*, and the choicy ruff *Seriolella porosa* Guichenot. Interestingly, sealworm larvae were not recorded, or have been reported in low intensities, in these fish species along the Patagonian coast ([14,49,55,64-66]; see Table 3). Moreover, sea lions consumed mainly smaller fish (< 35 cm TL), occasionally medium size fish (> 50 cm TL) and rarely large fish (> 65 cm TL) ([62,63]; N. A. García unpublished data). Therefore large fish, with higher densities of sealworm larvae, which could be considered as a potential route of transmission for sealworms, likely may act as a significant population ‘sink’ in the parasite life cycle, as they act as physical barrier for their transmission.

The present study suggests that the most important fish prey for sea lion in the Patagonian coast of Argentina, i.e. *E. anchoita*, *M. hubbsi* and *R. brasiliensis*[62,63], present low levels of sealworm infections. However, for sea lions, transmission and recruitment of sealworms would be ensured by the high quantity of ingested prey (especially in the case of *M. hubbsi*, see [62,63]), even when they are slightly infected. Nevertheless, sea lions could also acquire heavy sealworm infections sporadically by foraging on small benthic fish with high prevalences (e.g. *P. nudigula* represents the 0.29% of the percentage by number of the diet composition of sea lions according to Romero *et al*. [63]), or by occasionally preying large fish with high intensities of sealworm larvae. Finally we cannot exclude, to a lesser extent, direct transmission of sealworm larvae between invertebrate hosts to definitive hosts, as there also records of some species of invertebrates in the food habits of sea lions from Patagonia [62,63].

Distribution in fish host tissue also apparently differs between species of *Pseudoterranova*[42]. Recently, Kuhn *et al*. [67] recovered most of the third-stage larvae of *P. decipiens* (s.s.) from the muscle of *Osmerus eperlanus* L. from the North Sea. In the Southern Hemisphere, Palm [68] reported that the preferred site of infestation was the body cavity and the liver for sealworm larvae in fishes from the Antarctic waters. Our results suggest that the main microhabitat of sealworms in Patagonia is the muscle, and particularly those in the epaxial region for L3 of *P. cattani* infecting *P. nudigula*. One could wonder whether larval distribution could be affected by post-mortem migration of sealworms. However, this seems unlikely because all larvae were found encapsulated. On the other hand, according to the available evidence, apparently the main microhabitat of sealworms in South America is also the muscles; however, these tissues are not systematically analysed for parasites in this area see (Additional file 1: Table S1). This may result in the infection parameters of sealworm larvae in many studies being be underestimated. To date, several methods are available to detect larval sealworms in fish muscles [42], therefore we urge researchers to consider this location in further parasitological studies to provide complete information about the sealworm larvae in the marine fish helminth communities, which, moreover, will be particularly useful for stock discrimination studies.

## Conclusions

In conclusion, in this study we provided an overview of the current state of knowledge on the taxonomy and ecology of sealworm larvae in the southwestern Atlantic. The first molecular identification, description and microhabitat characterisation of sealworm larvae from the Patagonian coast of Argentina are provided. Additionally, we reported the infection levels of sealworms on 20 fish species in order to elucidate the life cycle of these nematodes in this area. We are aware that our ecological results convey a rather static picture of the dispersion of sealworm larvae for a short period of time. Furthermore, for some species of fish, few specimens could be collected and, therefore, sealworm larvae might not have been recorded due to low sample size. Further studies on sealworm from invertebrates, fish and pinnipeds hosts from this area are necessary to understand the systematic, biology and population dynamics of this nematode. However, this study provides a starting point to investigate the life cycle of sealworms in the Argentine Patagonian coast.

## Competing interests

The authors declare that they have no competing interests.

## Authors’ contributions

JSH-O and FEM conceived the study. JSH-O, NAG and EAC obtained the samples. JSH-O, MV-M and FEM undertook the morphological study. IB-C carried out the molecular analyses. JSH-O and FJA performed the statistical analyses. JSH-O, FJA, IB-C and FEM drafted the manuscript. EAC and JAR revised the manuscript. All authors read and approved the final manuscript.

## Supplementary Material

Additional file 1: Table S1 Check list of records of third-stage larvae of *Pseudoterranova* spp. in fishes from South America. *Abbreviations:* Bc, body cavity; Li, liver; MA, host muscles analysed for parasites; MH, microhabitat; Me, mesenteries; MS, mean number of sealworms; Mu, muscle; N.S., not specified; NI, number of infected hosts; TL, total length ± standard deviation (or range). Click here for file
