# Supplementary material for: Description, microhabitat selection and infection patterns of sealworm larvae (Pseudoterranova decipiens species complex, nematoda: ascaridoidea) in fishes from Patagonia, Argentina
Source: Parasit Vectors. 2013 Aug 29;6:252. doi: 10.1186/1756-3305-6-252 (PMC3847798; doi:10.1186/1756-3305-6-252)
Supplement: Additional file 1: Table S1 — Check list of records of third-stage larvae of Pseudoterranova spp. in fishes from South America. Abbreviations: Bc, body cavity; Li, liver; MA, host muscles analysed for parasites; MH, microhabitat; Me, mesenteries; MS, mean number of sealworms; Mu, muscle; N.S., not specified; NI, number of infected hosts; TL, total length ± standard deviation (or range). [file 1756-3305-6-252-S1.pdf]

Additional file 1 Check list of records of third-stage larvae of *Pseudoterranova* spp. in fishes from South America. Abbreviations: Bc, body cavity; Li, liver; MA, host muscles analysed for parasites; MH, microhabitat; Me, mesenteries; MS, mean number of sealworms; Mu, muscle; N.S., not specified; NI, number of infected hosts; TL, total length ± standard deviation (or range).

Order/Family	Species	Locality*	*n*	TL (cm)	MA	MH	NI	MS	References
**Anguilliformes**									
Congridae	*Conger orbignianus*	North Argentina[a]	50	–	Yes	Me	1	1	[1]
**Atheriniformes**									
Atherinopsidae	*Odontesthes nigricans*	Patagonia, Argentina[a]	125	(16.3–16.6)**	No	Bc	–	–	[2]
	Odontesthes smitti	Patagonia, Argentina[a]	118	(21.7–28.3)**	No	Bc	1	10	[3]
**Batrachoidiformes**									
Batrachoididae	*Aphos porosus*	Central Chile[b]	8	28.3 ± 1.5	N.S.	–	6	10	[4]
		Central Chile[a]	30	26.8 ± 3.9	Yes	Bc, Me, Mu	24	128	[5]
		South Chile[c]	13	–	Yes	Mu	6	10	[6]
	Porichthys porosissimus	North Argentina[a]	166	(11.5–29.7)	No	Bc	26	39	[7]
**Beloniformes**									
Belonidae	*Tylosurus acus acus*	Brazil[a]	31	(58.0–105.5)	N.S.	Me	3	4	[8]
**Gadiformes**									
Gadidae	*Micromesistius australis*	South Chile[a]	61	(26.0–57.0)	N.S.	–	3	4	[9]
Macrouridae	*Nezumia pulchella*	North Chile[a]	167	(26.6–32.1)**	No	–	2	2	[10]
		Central Chile[a]	50	31.1 ± 2.3	No	–	1	1	[10]
Merlucciidae	*Macruronus magellanicus*	South Chile[c]	288	–	Yes	Mu	9	29	[6]
		Central Chile[b]	3	134.3 ± 55.7	N.S.	–	1	1	[4]
		South Chile[b]	4	(22.0–28.0)	Yes	Mu	1	1	[11]
	Merluccius australis	South Chile[b]	685	(45.0–95.0)	No	Bc, Me, Mu	76	99	[12]
	Merluccius gayi gayi	Central Chile[b]	1051	(42.7–46.6)**	No	Me	211	897	[13]
		South Chile[b]	121	45.4 ± 10.1	No	Me	42	213	[13]
		South Chile[c]	34	–	Yes	Mu	19	71	[6]
		South Chile[b]	17	(36.0–50.0)	Yes	Mu	4	8	[11]
	Merluccius hubbsi	Patagonia, Argentina[a]	278	(38.7–40.4)**	No	Me	2	2	[14]
		Patagonia, Argentina[a]	42	(57.0–78.0)	Yes	Mu	4	8	[15]
**Mugiliformes**									
Mugilidae	*Mugil cephalus*	Central Chile[c]	107	(15.5–46.0)	No	Hi	1	1	[16]

**Ophidiiformes**									
Ophidiidae	*Genypertus* sp.	South Chile[c]	1	60	N.S.	Li, Me	–	–	[17]
	Genypterus blacodes	Argentina[b]	101	(24.0–127.0)	No	Me	4	20	[18]
		South Chile[c]	5	–	Yes	Mu	2	36	[6]
	Genypterus brasiliensis	Patagonia, Argentina[b]	31	(40.0–75.0)	No	Me	6	8	[18]***
		Brazil[a]	55	29.0–70.0	N.S.	Me	3	5	[19]***
		Brazil[b]	74	(41.5-93)	Yes	Me	4	5	[20]***
		Brazil[a]	74	(41.5-93)	Yes	Me	8	22	[20]***
	Genypterus chilensis	Central Chile[c]	80	(43.6-73.5)	Yes	Li, Mu	5	22	[21]
		South Chile[c]	7	–	Yes	Mu	4	27	[6]
		South Chile[b]	18	(41.0–55.0)	Yes	Mu	9	12	[11]
	Raneya brasiliensis	Patagonia, Argentina[a]	107	24.5 ± 1.7	Yes	–	1	1	[22]
**Perciformes**									
Blenniidae	*Hypsoblennius sordidus*	South Chile[a]	12	(4.8–10.2)	No	Mu	1	1	[23]
Carangidae	*Caranx hippos*	Brazil[a]	60	(27.0–64.0)	N.S.	–	6	9	[24]
	Caranx latus	Brazil[a]	55	(26.0–43.0)	N.S.	–	18	401	[24]
	Trachurus murphyi	North Chile[b]	600	36.3 ± 2.9	No	–	2	3	[25]
		Central Chile[b]	600	33.9 ± 2.3	No	–	17	28	[25]
		South Chile[c]	35	–	Yes	Mu	10	60	[6]
		South Chile[b]	16	(34.0–47.0)	Yes	Mu	5	12	[11]
		Chile–Oceanic[b]	183	43.2 ± 2.1	No	–	3	3	[25]
Pinguipedidae	*Pinguipes brasilianus*	Patagonia, Argentina[a]	102	(32.7–37.1)**	No	Me	5	7	[26]
	Pseudopercis semifasciata	North Argentina[a]	50	(67.5–71.2)**	No	Me	41	547	[27]
		Patagonia, Argentina[a]	50	67.2 ± 7.2	No	Me	38	220	[27]
Pomatomidae	*Pomatomus saltatrix*	Brazil[c]	55	(32.0–52.0)	N.S.	–	8	33	[28]
Priacanthidae	*Priacanthus arenatus*	Brazil[a]	58	(14.0–54.0)	N.S.	–	5	23	[29]
Sciaenidae	*Cilus gilbert*	Central Chile[d]	57	(51.6–75.5)	No	–	36	153	[30]
		Central Chile[d]	29	(32.5–75.0)	No	–	23	139	[30]
		South Chile[c]	11	–	Yes	Mu	6	15	[6]
	Micropogonias furnieri	Brazil[a]	100	(10.0–66.0)	N.S.	Me	2	3	[31]
Scombridae	*Scomber japonicus*	North Argentina[a]	173	(29.7–53.4)	No	Me	13	4	[32]
		Brazil[c]	50	(20.0–25.0)	No	Me	5	–	[33]
Sparidae	*Pagrus pagrus*	Brazil[a]	90	(16.0–50.0)	N.S.	Me	6	15	[34]

Trichiuridae	*Trichiurus lepturus*	Brazil[a]	55	(108.0–148.0)	N.S.	–	21	46	[35]
**Pleuronectiformes**									
Paralichthyidae	*Hippoglossina macrops*	South Chile[c]	21	–	Yes	Mu	3	4	[6]
	Hippoglossina montemaris	South Chile[c]	8	–	Yes	Mu	1	1	[6]
	Paralichthys adspersus	North Chile[b]	179	(31.2–34.2)	Yes	Bc, Mu	16	47	[36]
	Paralichthys isosceles	North Argentina[a]	51	28.0 ± 2.1	Yes	Me, Mu	1	1	[37]
	Paralichthys microps	South Chile[b]	10	(26.0–38.0)	Yes	Mu	7	14	[11]
	Paralichthys patagonicus	North Argentina[a]	51	35.2 ± 2.6	Yes	Me, Mu	9	13	[37]
	Xystreurys rasile	North Argentina[a]	48	29.3 ± 2.6	Yes	Me. Mu	1	3	[37]
**Scorpaeniformes**									
Normanichthyidae	*Normanichthys crockeri*	South Chile[a]	11	9.0 ± 0.2	No	Mu	1	1	[23]
Sebastidae	*Helicolenus lengerichi*	Central Chile[a]	30	28.1 ± 4.5	No	–	1	1	[38]
		Central Chile[a]	56	30.9 ± 2.3	No	–	2	2	[39]
	Sebastes capensis	Central Chile[a]	42	(27.5–27.8)**	No	–	10	13	[39]

*Specific identification of sealworms larvae: [a]*Pseudoterranova* sp.; [b]*P. decipiens*; [c]*Phocanema* sp.; [d]*P. cattani.*

**Range calculated from host total length means.

***Fish identified as *Genypterus brasiliensis* by the authors.

References

[1] Timi JT, Lanfranchi AL: **Ontogenetic changes in heterogeneity of parasite communities of fish: disentangling the relative role of compositional versus abundance variability.** *Parasitology* 2013, **140:**309-317.

[2] Carballo MC, Navone GT, Cremonte F: **Parasites of the silversides *Odontesthes smitti* and *Odontesthes nigricans* (Pisces: Atherinopsidae) from Argentinean Patagonia.** *Comp Parasitol* 2011, **78:**95-103.

[3] Carballo MC, Cremonte F, Navone GT, Timi JT: **Similarity in parasite community structure may be used to trace latitudinal migrations of *Odontesthes smitti* along Argentinean coasts.** *J Fish Biol* 2012, **80:**15-28.

[4] Torres P, Contreras A, Revenga J, Fritz N: **Helminth parasites in fishes from Valdivia and Tornagaleones river estuaries in the south of Chile.** *Mem I Oswaldo Cruz* 1993, **88:**491-492.

[5] Cortés Y, Muñoz G: **Infracomunidades de parásitos eumetazoos del bagre de mar *Aphos porosus* (Valenciennes, 1837) (Actinopterygii: Batrachoidiformes) en Chile central.** *Rev Biol Mar Oceanogr* 2008, **43:**255-263.

[6] Torres P, Hernández E, Sandoval I: **Anisakiasis and Phocanemiasis in marine fishes from the south of Chile.** *Int J Zoonoses* 1983, **10:**146-150.

[7] Tanzola RD, Guagliardo SE, Brizzola SM, Arias MV: **Helminth fauna of *Porichthys porosissimus* (Pisces: Batrachoidiformes) in the estuary of Bahia Blanca Argentina.** *Helminthologia* 1997, **34:**221-227.

[8] Tavares LER, Bicudo AJA, Luque JL: **Metazoários parasitos do agulhão *Tylosurus acus* (Lacépède, 1803) (Osteichthyes: Belonidae) do litoral do estado do Rio de Janeiro, Brasil.** *Rev Bras Parasitol Vet* 2004, **13:**36-40.

[9] Chávez RA, González MT, Oliva ME, Valdivia IM: **Endoparasite fauna of five Gadiformes fish species from the coast of Chile: host ecology versus phylogeny.** *J Helminthol* 2012, **86:**10-15.

[10] Salinas X, González MT, Acuña E: **Metazoan parasites of the thumb grenadier *Nezumia pulchella*, from the south-eastern Pacific, off Chile, and their use for discrimination of host populations.** *J Fish Biol* 2008, **73:**683-691.

[11] Torres P, Moya R, Lamilla J: **Nematodos anisákidos de interés en salud pública en peces comercializados en Valdivia, Chile.** Arch Med Vet 2000, **32:**107-113.

[12] George-Nascimento M, Arancibia H: **La fauna parasitaria y la morfometría de la merluza austral *Merluccius australis* (Hutton) como indicadoras de unidades de stock.** *Biol Pesq* 1994, **23:**31-47.

[13] George-Nascimento M: **Populations and assemblages of parasites in hake, *Merluccius gayi*, from southeastern Pacific Ocean: Stock implications.** *J Fish Biol* 1996, **48:**557-568.

[14] Sardella NH, Timi JT: **Parasites of Argentine hake in the Argentine sea: population and infracommunity structure as evidence for host stock discrimination.** *J Fish Biol* 2004, **65:**1472-1488.

[15] Herreras MV, Aznar FJ, Balbuena JA, Raga JA: **Anisakid larvae in the musculature of the Argentinean hake, *Merluccius hubbsi*.** *J Food Protect* 2000, **63:**1141-1143.

[16] Fernández J: **Los parásitos de la lisa *Mugil cephalus* L., en Chile: sistemática y aspectos poblacionales (Perciformes: Mugilidae).** *Gayana, Zool* 1987, **51:**3-58.

[17] Torres P, González H: **Determinación de larvas de *Terranova* (=*Phocanema*) y *Anisakis* en *Genypterus* sp. Aspectos morfométricos e histopatológicos a nivel hepático.** *Bol Chil Parasitol* 1978, **33:**82-86.
[18] Sardella NH, Avedaño MF, Timi JT: **Parasite communities of *Genypterus blacodes* and *Genypterus brasiliensis* (Pisces: Ophidiidae) from Argentina.** *Helminthologia* 1998, **35:**209-218.
[19] Alves DR, Luque JL, Paraguassú AR: **Community ecology of the metazoan parasites of pink cusk-eel, *Genypterus brasiliensis* (Osteichthyes: Ophidiidae), from the coastal zone of the state of Rio de Janeiro, Brazil.** *Mem I Oswaldo Cruz* 2002, **97:**683-689.
[20] Knoff M, São Clemente SC, Fonseca MCG, Andrada CG, Padovani RES, Gomes DC: **Anisakidae parasitos de congro-rosa, *Genypterus brasiliensis* Regan, 1903 comercializados no estado do Rio de Janeiro, Brasil de intersse na saúde pública.** *Parasitol Latinoam* 2007, **62:**127-133.
[21] Vergara LA, George-Nascimento M: **Contribución al estudio del parasitismo en el congrio colorado *Genypterus chilensis* (Guichenot, 1848).** *Bol Chil Parasitol* 1982, **37:**9-14.
[22] Vales DG, García NA, Crespo EA, Timi JT: **Parasites of a marine benthic fish in the Southwestern Atlantic: searching for geographical recurrent patterns of community structure.** *Parasitol Res* 2011, **108:**261-272.
[23] Sepúlveda F, Marín SL, Carvajal J: **Metazoan parasites in wild fish and farmed salmon from aquaculture sites in southern Chile.** *Aquaculture* 2004, **235:**89-100.
[24] Luque JL, Alves DR: **Ecologia das comunidades de metazoários parasitos, do xaréu, *Caranx hippos* (Linnaeus) e do xerelete, *Caranx latus* Agassiz (Osteichthyes, Carangidae) do litoral do estado do Rio de Janeior, Brasil.** *Rev Bras Zool* 2001, **18:**399-410.
[25] George-Nascimento M, Arancibia H: **Stocks ecológicos del jurel (*Trachurus symmetricus murphyi* Nichols) en tres zonas de pesca frente a Chile, detectados mediante comparación de su fauna parasitaria y morfometría.** *Rev Chil Hist Nat* 1992, **65:**453-70.
[26] Timi JT, Lanfranchi AL, Etchegoin JA, Cremonte F: **Parasites of the Brazilian sandperch *Pinguipes brasilianus* Cuvier: a tool for stock discrimination in the Argentine Sea.** *J Fish Biol* 2008, **72:**1332-1342.
[27] Timi JT, Lanfranchi AL: **The metazoan parasite communities of the Argentinean sandperch *Pseudopercis semifasciata* (Pisces: Perciformes) and their use to elucidate the stock structures of the host.** *Parasitology* 2009, **136:**1209-1219.
[28] Luque JL, Chaves ND: **Ecologia da comunidade de metazoários parasitos da anchova *Pomatomus saltator* (Linnaeus) (Osteichthyes, Pomatomidae) do litoral do estado do Rio de Janeiro, Brasil.** *Rev Brasil Zool* 1999, **16:**711-723.
[29] Tavares LER, Luque JL, Neto SLB: **Ecologia da comunidade de metazoários parasitos do olho-de cão *Priacanthus arenatus* (Cuvier, 1829) (Osteichthyes, Priacanthidae) do litoral do estado do Rio de Janeiro, Brasil.** *Rev Brasil Zoociênc* 2001, **3:**45-59.
[30] Garcías F, Mendoza R, George-Nascimento M: **Variación entre años de las infracomunidades de parásitos metazoos de la corvine *Cilus gilberti* (Pisces: Sciaenidae) en Chile.** *Rev Chil Hist Nat* 2001, **7:**833-840.
[31] Alves DR, Luque JL: **Community ecology of the metazoan parasites of white croaker, *Micropogonias furnieri* (Osteichthyes: Sciaenidae), from the coastal zone of the state of Rio de Janeiro, Brazil.** *Mem I Oswaldo Cruz* 2001, **96:**145-53.

[32] Cremonte F, Sardela NH: **The parasito fauna of *Scomber japonicus* Houttuyn, 1782 (Pisces: Scombridae) in two zones of the Argentine sea.** Fish Res 1997, **31:**1-9.
[33] Rego AA, Santos CP: **Helmintofauna de cavalas, *Scomber japonicus* Houtt, do Rio de Janeiro.** *Mem I Oswaldo Cruz* 1983, **78:**443-48.
[34] Paraguassú AR, Luque JL, Alves DR: **Community ecology of the metazoan parasites of red porgy, *Pagrus pagrus* (L., 1758) (Osteichthyes, Sparidae), from the coastal zone, State of Rio de Janeiro, Brazil.** *Maringá* 2002, **24:**461-467.
[35] Silva LO, Luque JL, Alves DR, Paraguassú AR: **Ecologia da comunidade de metazoários parasitos do peixe-espada *Trichiurus lepturus* Linnaeus (Osteichthyes, Trichiuridae) do litoral do estado do Rio de Janeiro, Brasil.** *Rev Brasil Zoociênc* 2000, **2:**115-133.
[36] Oliva ME, Castro RE, Burgos R: **Parasites of the flatfish *Paralichthys adspersus* (Steindachner, 1867) (Pleuronectiformes) from Northern Chile.** *Mem I Oswaldo Cruz* 1996, **91:**301-306.
[37] Alarcos AJ, Timi JT: **Parasite communities in three sympatric flounder species (Pleuronectiformes: Paralichthyidae).** *Parasitol Res* 2012, **110:**2155-2166.
[38] George-Nascimento M, Iriarte JL: **Las infracomunidades de parásitos metazoos del chancharro *Helicolenus lengerichi* Norman, 1937 (Pisces, Scorpaenidae): un ensamble no interactive de especies.** *Rev Chil Hist Nat* 1989, **62:**217-27.
[39] Balboa L, George-Nascimento M: **Variaciones ontogenéticas y entre años en las infracomunidades de parásitos metazoos de dos especies de peces marinos de Chile.** *Rev Chil Hist Nat* 1998, **71:**27-37.
